# Enhancing resolution with the extended image restoration method: strain field energy and correlation length analysis in Bragg coherent X-ray diffraction imaging

**DOI:** 10.1107/S1600577525002942

**Published:** 2025-04-25

**Authors:** Kyuseok Yun, Sungwook Choi, Hyunjung Kim

**Affiliations:** a Center for Ultrafast Phase Transformation, Department of Physics Sogang University Seoul04107 Republic of Korea; Tohoku University, Japan

**Keywords:** Bragg coherent X-ray diffraction imaging, imaging upsampling, extended image restoration, strain field energy, correlation length

## Abstract

Understanding atomic-level imperfections is crucial in various technological applications. Bragg coherent X-ray diffraction imaging (BCDI) enables non-destructive, three-dimensional imaging of those materials under *in situ* and *operando* conditions but has limited spatial resolution. This limitation hinders accurate calculations of physical quantities, *e.g.* strain field energy and strain correlation lengths. In this study, we introduce the extended image restoration (ExImRes) method, which infers enhanced resolution images based primarily on the process of averaging and combining multiple datasets obtained by restricting the original measured datasets through binning or cropping. We apply ExImRes to two nanocrystal examples—a chiral gold nanoparticle and a platinum nanoparticle—with an improved spatial resolution that allowed us to obtain precise calculation results of strain field energy and the correlation lengths of atomic deformations. The enhanced images reveal detailed lattice-scale information previously inaccessible through traditional BCDI methods. Our findings advance ExImRes to obtain high-resolution analysis in imaging techniques that involve reciprocal to real space transformations and understand underlying phenomena in materials science.

## Abstract 2

The Extended Image Restoration (ExImRes) method has been developed to enhance the spatial resolution of Bragg coherent X-ray diffraction imaging by combining multiple lower-resolution images from the same dataset. Applied to chiral gold and platinum nanoparticles as examples, ExImRes overcomes traditional resolution limits to enable precise calculations of strain field energy and atomic deformation correlation lengths, revealing detailed lattice-scale information previously inaccessible.

## 1. Introduction

Gaining insight into atomic-level phenomena in crystalline materials—such as strain, defects and dislocations—is essential for optimizing their mechanical, electrical and optical properties. High-resolution imaging techniques have been pivotal in exploring these phenomena, with electron microscopy standing out as a compelling method (Spence, 2013 ▸). Electron microscopy techniques, including transmission electron microscopy (TEM) and scanning transmission electron microscopy (STEM), enable direct visualization of atomic arrangements, providing insights into lattice structures and defect formations at sub-angstrom resolutions (Kabius *et al.*, 2009 ▸; Phillips *et al.*, 2012 ▸). However, these methods have inherent limitations, such as complex sample preparation, susceptibility to radiation damage and limited penetration depth, which can impede accurate analysis of thick or beam-sensitive materials.

Bragg coherent X-ray diffraction imaging (BCDI) offers the advantage of being able to non-destructively measure the distortion map inside a crystal, a feature that stands out prominently among imaging techniques utilizing coherent X-ray beams produced by advanced synchrotron sources. BCDI offers a complementary approach to electron microscopy by enabling non-destructive, three-dimensional imaging of crystalline materials under realistic environmental conditions. However, the resolution of BCDI, typically <10 nm, is not yet far behind electron microscopy (Robinson & Harder, 2009 ▸; Cha *et al.*, 2013 ▸). The fundamental principle of BCDI involves measuring the coherent diffraction pattern around a Bragg peak, which contains both amplitude and phase information related to the electron density distribution and displacement field within the crystal, respectively. By employing iterative phase retrieval algorithms, BCDI reconstructs real-space images that reveal atomic displacements within the crystal lattice with picometre-scale resolution. This capability allows for detailed mapping of strain fields and the identification of defects at the nanoscale (Ulvestad *et al.*, 2015 ▸; Kim *et al.*, 2018 ▸; Carnis *et al.*, 2019 ▸; Kim *et al.*, 2019 ▸; Choi *et al.*, 2020 ▸; Kang *et al.*, 2020 ▸; Choi *et al.*, 2023 ▸).

To achieve higher spatial resolution in BCDI, it is essential to cover a broader range of reciprocal space, allowing for the reconstruction of finer details in the real-space image. However, practical limitations such as signal-to-noise ratios, finite detector sizes and the dynamic range constraints of pixelated detectors hinder the collection of diffraction data beyond technical limits. These hurdles prevent the acquisition of high-resolution data, resulting in images that average over multiple atomic layers and thus have limited spatial resolution. For BCDI with an energy higher than 50 keV, where it initially does not meet the Nyquist–Shannon criterion, the upsampling method enhances the density of data points in reciprocal space, enabling sufficient sampling and thereby making the generation of coherent diffraction images feasible (Maddali *et al.*, 2019 ▸). Methods have also been devised to improve the resolution of BCDI results under given technical limitations by attempting phase retrieval after upsampling the diffraction pattern (Chushkin & Zontone, 2013 ▸).

Consequently, the physical quantities from BCDI are providing relative values. A representative example is strain field energy (Anderson *et al.*, 2017 ▸). The limited spatial resolution impedes the accurate calculation of strain distributions within the crystal lattice. This nature poses challenges for precisely characterizing material properties at the nanoscale (Karpov & Fohtung, 2019 ▸; Kim *et al.*, 2019 ▸).

In this study, we address developing the extended image restoration (ExImRes) technique, an advanced image upsampling method, to enhance resolution by combining multiple lower-resolution images, thereby enabling the calculation of the strain field energy and the atomic deformation correlation length. ExImRes enables us to surpass the experimental resolution and to extract finer details. As an application of the ExImRes method, we obtain accurate values of strain field energy and the correlation length (strain propagation length) of atomic deformations. We apply this method to a few nanocrystal samples, including a chiral gold nanoparticle and a platinum (Pt) nanoparticle, demonstrating its potential to deliver atomic-scale insights previously in­accessible through traditional BCDI methods.

## 2. Methodology

### 2.1. Overall process of ExImRes

**Figure 1:**
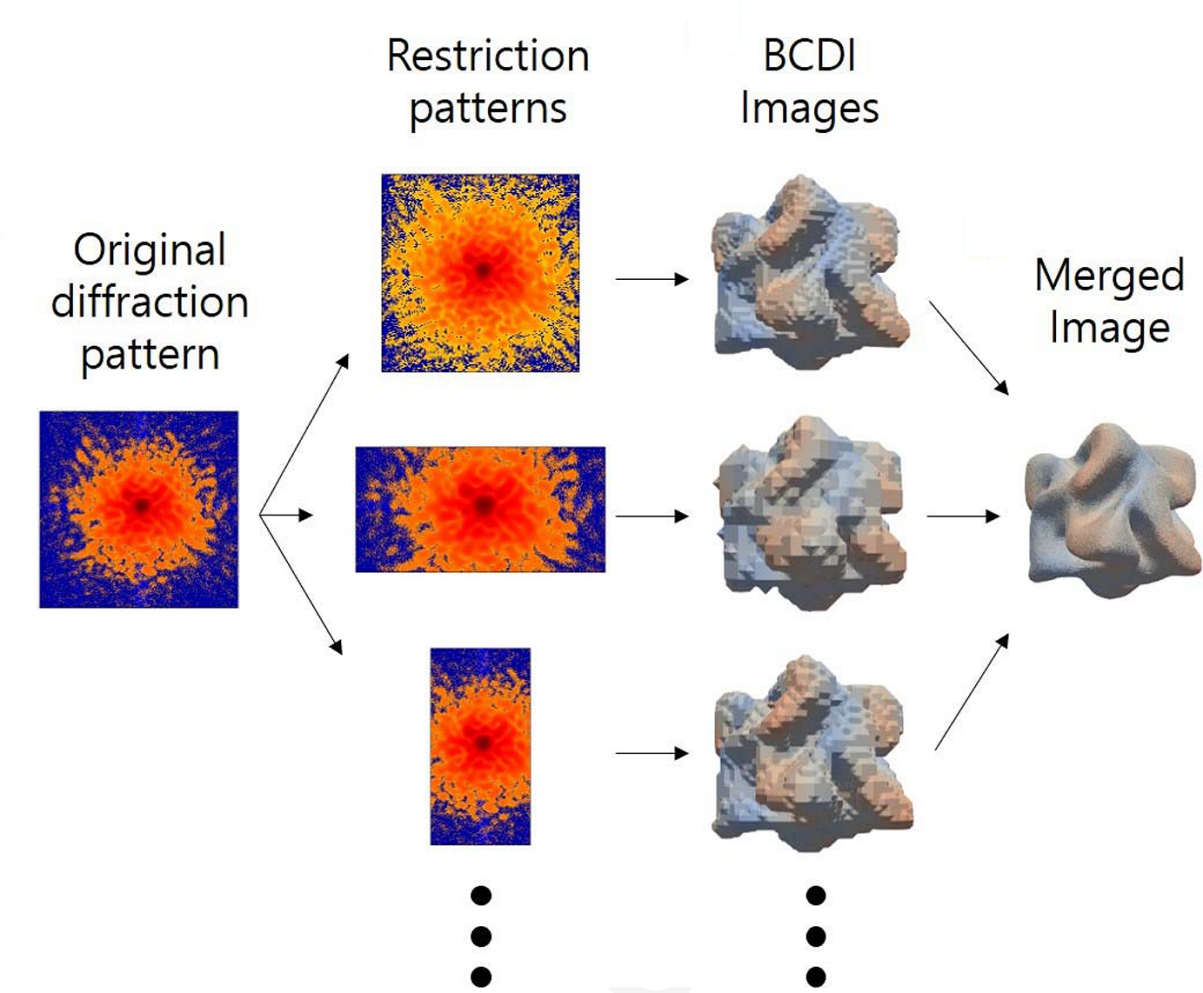
A schematic for the ExImRes. ExImRes is a method that involves (*a*) obtaining the original diffraction pattern from an experiment, (*b*) generating a number of restricted patterns through processes such as binning or cropping, (*c*) performing phase reconstruction on each of these restricted patterns to produce individual BCDI images, and (*d*) merging these images to achieve an enhanced resolution image.

Fig. 1 ▸ illustrates a schematic overview of the ExImRes process. The ExImRes method is developed to enhance the spatial resolution of BCDI beyond technical limitations. While it is impossible to achieve a higher resolution than the image obtained directly from experimental data, ExImRes leverages the generation of multiple lower-resolution images from the same dataset. By combining these images, we can extract finer details corresponding to differences in voxel sizes, effectively surpassing the original resolution limitations. We applied ExImRes to two nanocrystal samples—a chiral gold nanoparticle and a Pt nanoparticle—to demonstrate its capability to enhance image resolution and enable more accurate calculations of strain field energy and atomic deformation correlation lengths.

To estimate resolution, we calculate the phase retrieval transfer function (PRTF) and the point spread function (PSF) (Cherukara *et al.*, 2018 ▸). The plots of the PRTF are shown in Fig. S1 of the supporting information. The PRTF without ExImRes was obtained from the average of ten independent phase reconstructions. Whereas the PRTF of the Pt sample with ExImRes showed an improvement in resolution, the PRTF of the chiral gold sample did not fall below the threshold value of 1/e, making it impossible to define the resolution. This means that, not only were the phases in all regions of the measured pattern consistently reconstructed, but also that consistent information was retrieved even in regions with very low intensity within the measured pattern, leading to an overall resolution that surpasses that of the measured pattern. Fig. S2 of the supporting information presents the reciprocal space data used for the PRTF calculation: the pattern from the measured data, the averaged pattern of the ten Fourier transformed from independent phase retrieval results and the averaged Fourier transforms of the images just before merging in the ExImRes method. For the PSF, the spatial resolution was determined by the full width at half-maximum (FWHM) of the function obtained through deconvolution based on the imaging results from a perfect crystal. The results of the 3D PSF by blind deconvolution method are shown in Fig. S3 of the supporting information.

### 2.2. Generating multiple datasets and phase retrieval processing

**Figure 2:**
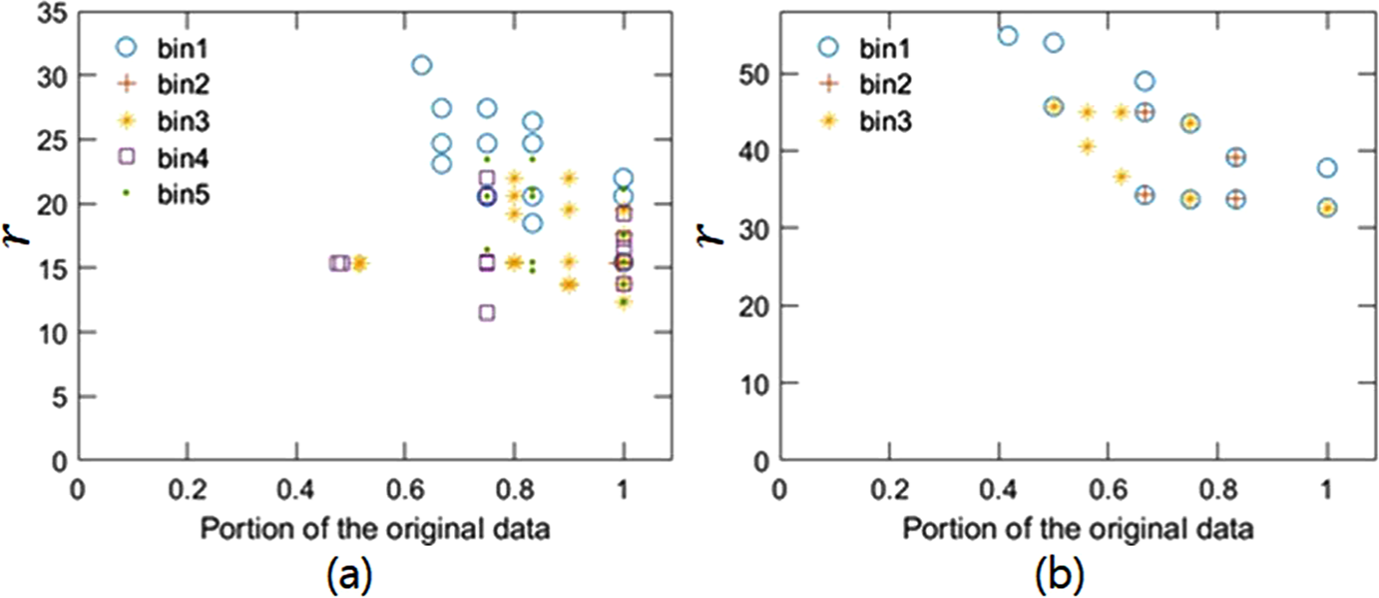
The relationship between *r*, the ratio of the voxel size of the lattice constant, and the amount of cropping. Panels (*a*) and (*b*) represent the results from the chiral gold particle and the Pt nanoparticle, respectively. The chiral gold particle underwent binning from 1 to 5, whereas the Pt nanoparticle underwent binning from 1 to 3, both of which were achievable within the range that satisfies the Nyquist–Shannon sampling ratio.

To implement ExImRes, we generated multiple image reconstructions with different voxel sizes from a single experimental dataset. This was achieved by modifying the diffraction patterns through methods such as binning the detector data or cropping the outer portions of the diffraction patterns. In the detector plane, the data were sufficiently oversampled, providing flexibility for data manipulation like binning and cropping. However, in the rocking curve direction, the step size was not fine enough for effective binning or frame skipping. Despite this, the overall range of the rocking curve was sufficiently extensive to enable frame cropping. These modifications result in datasets that satisfy the Nyquist–Shannon sampling theorem to varying degrees, allowing for image reconstructions at different resolutions. The phase retrieval process was conducted using a MATLAB-based phase retrieval algorithm, which was built based on the work of Fienup (1982 ▸) and Clark *et al.* (2013 ▸), combining the ER and HIO algorithms in a 20:180 ratio, with a total of 620 iterations. In particular, we applied these methods to the datasets of the chiral gold nanoparticle and the Pt nanoparticle, obtaining 114 and 38 sets of image reconstructions, respectively. Fig. 2 ▸ shows the change in resolution based on binning and cropping.

### 2.3. Upsampling and alignment

The individual imaging results are upsampled to achieve a consistent voxel size using cubic spline interpolation (De Boor, 1978 ▸). Although cubic spline interpolation is not strictly necessary due to the overall monotonic increase or decrease of the phase across the particle, it was chosen to minimize potential errors at local points in the phase that could arise from linear interpolation. Spatial alignment is then performed by centering the mass of the sample within the imaging array. This alignment is crucial to ensure consistency across different datasets, as it prevents errors caused by misaligned or flipped images. Interpolation methods are applied during upsampling to standardize voxel sizes before merging. In BCDI imaging results, the phase is not an absolute value but a relative one, making phase synchronization essential during alignment. To synchronize the phases of each image, we applied a phase translation to ensure that the total phase inside the crystal summed to zero. To aid understanding of this workflow, Fig. S4 of the supporting information illustrates how the process prior to merging follows the sequential steps of restriction, imaging and upsampling.

### 2.4. Averaging amplitudes and median of phases

In the merging process, amplitudes at corresponding voxel positions across all images are averaged, whereas phases are combined using a median. Averaging the amplitudes serves to confirm proper alignment by observing the overall amplitude distribution. Using the median for phases helps reduce the impact of outliers, particularly those located on the crystal’s surface.

### 2.5. Balancing errors through multiple images

Since each voxel represents an average of the atomic structure within, upsampling can introduce inaccuracies by either overestimating or underestimating values. However, combining a sufficient number of images helps balance these errors, as discrepancies at specific voxel locations tend to cancel each other out across the dataset. This process leads to a normal distribution of voxel values centered around a reliable mean.

### 2.6. Improved resolution via statistical averaging

By focusing on the mean values from the normal distribution obtained through multiple merged images, the resolution is enhanced. This statistical averaging sharpens the image by refining voxel values, ultimately providing a more accurate and high-resolution representation of the sample’s internal structure. This improved resolution allows for more precise calculations of properties such as strain field energy and atomic deformation correlation lengths.

### 2.7. Summary

The ExImRes method improved image resolution through a systematic approach involving multiple steps that revolve around averaging and combining restricted data sets. We summarize how it works as follows. First, multiple restricted data sets are generated by modifying the original data through techniques such as binning or cropping. These data sets may have different resolutions, which can be interpolated in later stages. Each data set is then processed individually using a phase retrieval algorithm, yielding complex imaging results where the amplitude indicates electron density and the phase represents atomic displacement. The resulting images are upsampled to the desired voxel size, and alignment is ensured by centering the mass of the sample within the imaging array for consistency across all data sets. In the next step, amplitudes at corresponding positions across images are averaged. At the same time, phases are combined using a median approach to reduce the impact of outliers, especially at the crystal surface. This process confirms proper alignment and balances errors since combining multiple images helps cancel out voxel-specific inaccuracies. Over time, these errors form a normal distribution of values around a mean. Finally, statistical averaging of these values refines the image by sharpening voxel data, ultimately enhancing resolution and providing a more accurate representation of the sample.

## 3. Results and discussion

### 3.1. Application of ExImRes to model datasets

To validate the effectiveness of the ExImRes method, we applied it to two different nanocrystal samples: a chiral gold nanoparticle and a Pt nanoparticle. The chiral gold nanoparticles were synthesized through enantioselective interactions between chiral ligands and gold surfaces, resulting in complex three-dimensional shapes (Lee *et al.*, 2018 ▸; Choi *et al.*, 2023 ▸). These intricate structures served as an ideal test case to assess whether ExImRes could accurately reconstruct complex geometries and subtle shapes at high resolutions. From a measured data resolution of 7.8 nm, we were able to achieve a best resolution of 4.2 nm in the case of *r* = 2, demonstrating the method’s capability to enhance resolution significantly. The detailed information on the parameters is shown in Table S1 of the supporting information.

After successfully demonstrating ExImRes on the chiral gold nanoparticles, we extended the application to a Pt nanoparticle sample. The Pt nanoparticle, formed via a dewetting process on a silicon wafer substrate, exhibited a relatively simple geometry with well defined facets (Kim *et al.*, 2018 ▸). By testing ExImRes on both nanostructured samples, we demonstrate the versatility and robustness of the method in enhancing image resolution across different material systems. The measured data resolution of the Pt nanoparticle was 18.1 nm, but we were able to reduce it to a best resolution of 9.1 nm in the case of *r* = 2.

### 3.2. Results for chiral gold nanoparticle

The BCDI pattern of the chiral gold nanoparticle was measured at the P10 beamline of PETRA III, achieving a voxel size of 6.9 nm. Using phase retrieval algorithms with various constraints—such as binning and cropping of the diffraction patterns—we generated 114 images with different conditions (detailed in Table S2). We introduced a parameter *r*, defined as the ratio of the voxel size to the lattice constant, to quantify the resolution of the resulting images. An *r* value of 1 indicates that the voxel size equals the lattice constant, while *r* = 5 implies that one voxel represents 5^3^ = 125 lattice units.

The chiral gold nanoparticle exhibited significant atomic displacements in certain regions, causing the phase range in some voxels to exceed the principal value range of −π to +π. To address this, we applied phase unwrapping corrections to ensure an accurate representation of the displacement fields. After correction, ExImRes enabled us to obtain high-resolution images for *r* values ranging from 2 to 50.

**Figure 3:**
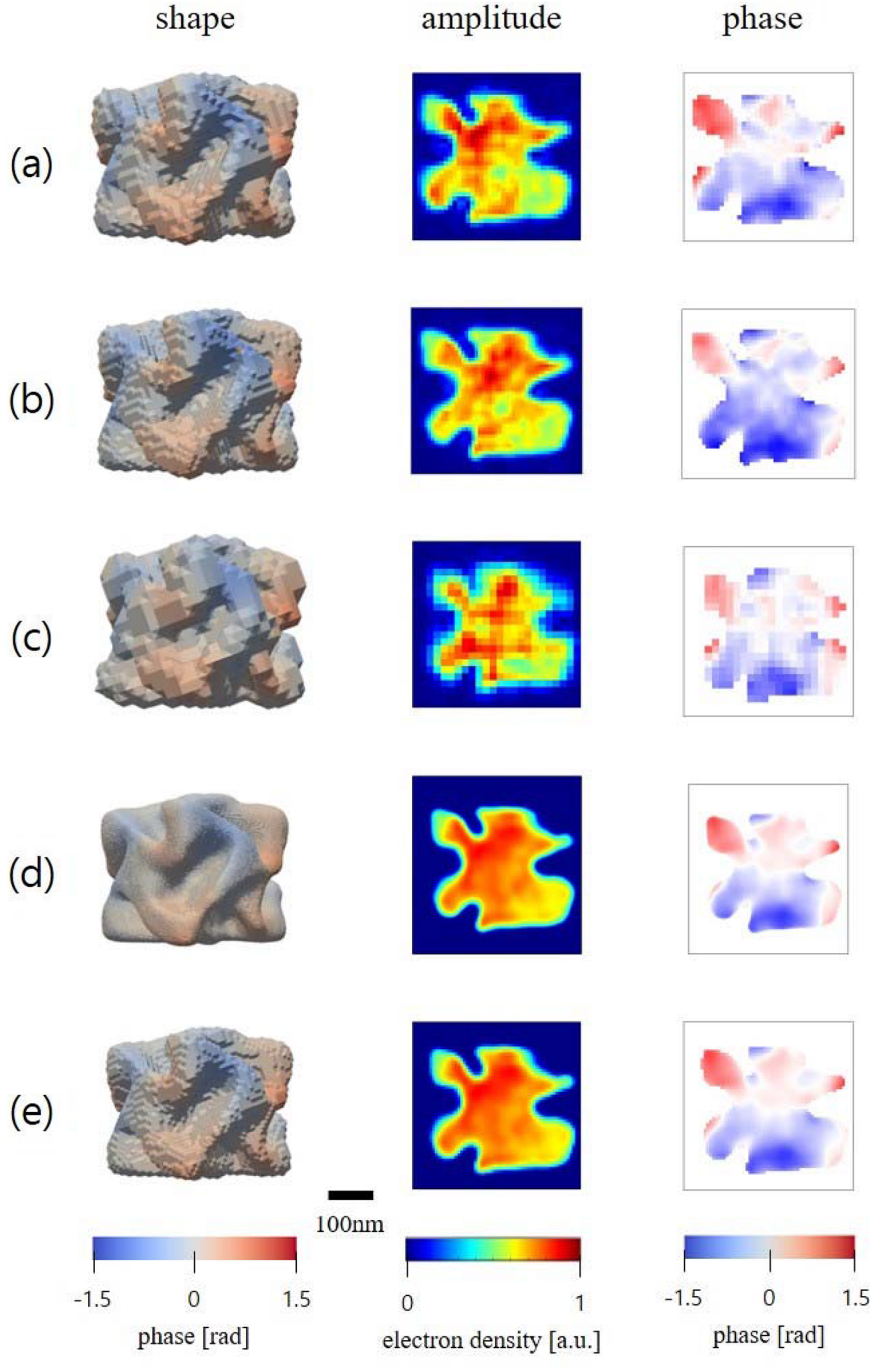
The shapes, amplitudes and phases of the center frames are represented for (*a*) the original experimental condition, (*b*) an example of binning, (*c*) an example of cropping, and (*d*) the merged results of *r* = 2 and (*e*) *r* = 10 for the chiral gold particle. The spatial resolutions were 7.8 nm, 8.2 nm, 13.3 nm, 4.2 nm and 5.3 nm, respectively.

Fig. 3 ▸ compares the reconstruction results before and after applying ExImRes. Figs. 3 ▸(*a*), 3 ▸(*b*) and 3(*c*) show individual reconstructions under original experimental conditions, with binning applied and with cropping applied, respectively. In the binned data shown in Fig. 3 ▸(*b*), although the reciprocal space coverage was similar to the original experimental condition, the reduced data size led to challenges in achieving consistent reconstructions upon repeated imaging. The cropped data in Fig. 3 ▸(*c*) resulted in reduced reciprocal space coverage, increasing the *r* value and thus degrading the resolution.

Figs. 3 ▸(*d*) and 3 ▸(*e*) present the ExImRes reconstructions for *r* = 2 and *r* = 10, respectively. The enhanced images reveal the curved shapes of the chiral gold nanoparticle with improved clarity, and the phase distributions exhibit smooth transitions without artifacts. Notably, despite differences in resolution due to varying *r* values, both reconstructions maintain consistent overall shape and phase information. This consistency demonstrates that ExImRes effectively enhances resolution while preserving the intrinsic structural and displacement characteristics of the sample.

### 3.3. Results for platinum nanoparticle

The BCDI pattern of the Pt nanoparticle was also measured at the P10 beamline of PETRA III, achieving a voxel size of 12.8 nm. We generated 38 images using different constraints on the diffraction patterns (detailed in Table S3). The Pt nanoparticle exhibited well defined shapes of its facets, resulting from the dewetting process during sample preparation. However, unless the voxel grid was perfectly aligned with the crystallographic facets, a stepped appearance was observed on the facet surfaces in the reconstructions.

**Figure 4:**
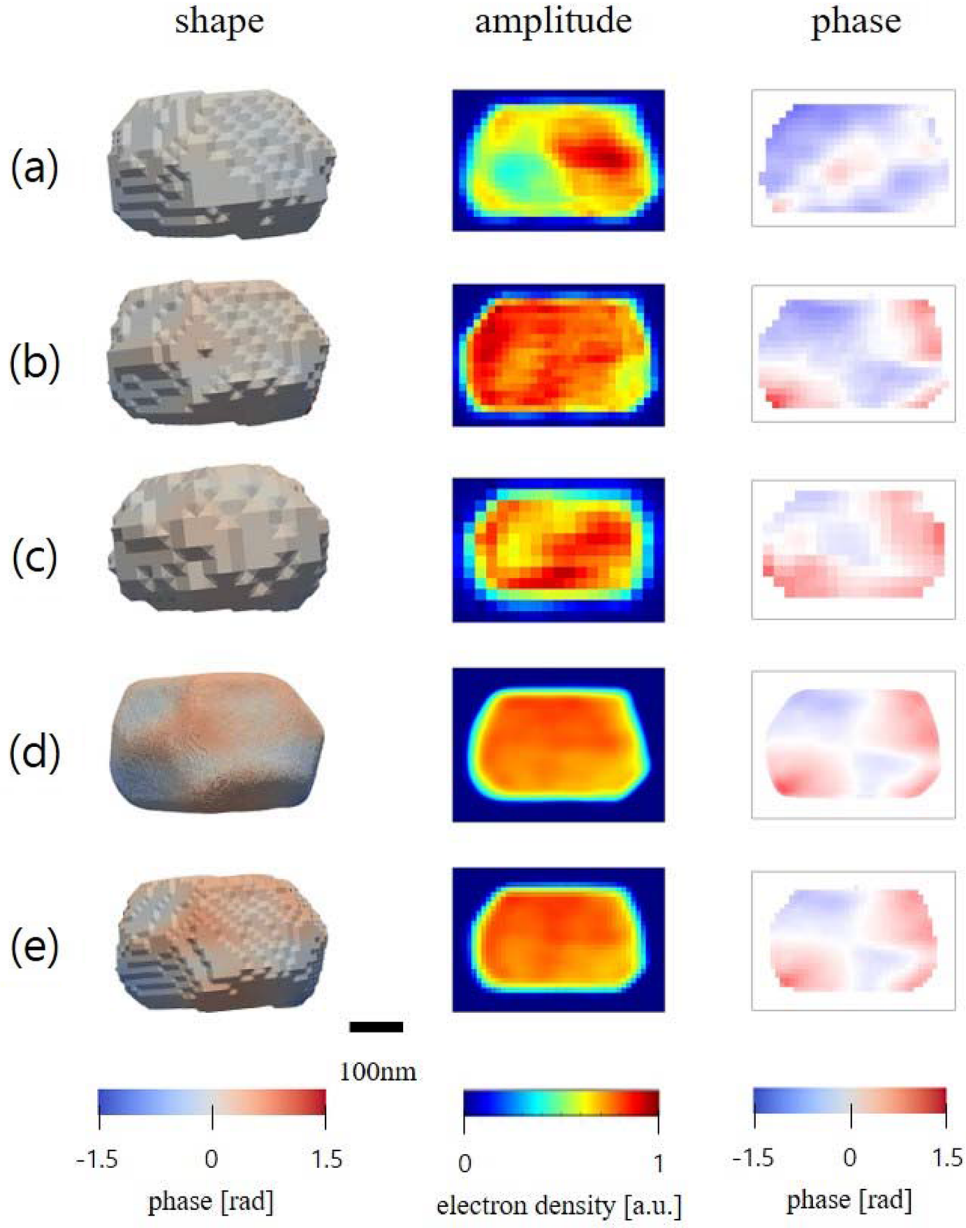
The shapes, amplitudes and phases of the center frames are represented for (*a*) the original experimental condition, (*b*) an example of binning, (*c*) an example of cropping, and (*d*) the merged results of *r* = 3 and (*e*) *r* = 25 for the Pt nanoparticle. The spatial resolutions were 18.1 nm, 21.7 nm, 36.5 nm, 9.1 nm and 16.2 nm, respectively.

ExImRes was successfully applied to the Pt nanoparticle data, as illustrated in Fig. 4 ▸. Figs. 4 ▸(*a*), 4 ▸(*b*) and 4(*c*) display individual reconstructions under original experimental conditions, with binning and with cropping, respectively. It is important to note that the phase values, representing atomic displacements, are relative quantities. Therefore, individual reconstructions may exhibit shifted phase distributions, but the relative phase differences remain consistent across the images.

The merged ExImRes reconstructions for *r* = 3 and *r* = 25 are presented in Figs. 4 ▸(*d*) and 4 ▸(*e*), respectively. The enhanced images display consistent shape and phase information throughout the entire nanoparticle. In particular, the reconstruction at *r* = 3 clearly reveals the well defined facets of the Pt nanoparticle, indicating that ExImRes effectively enhanced the spatial resolution and accurately captured the geometry of the nanoparticle.

### 3.4. Strain field energy calculations

By applying the ExImRes method, we obtained high-resolution images that accurately represent the atomic displacements at precise positions within the nanoparticles. This detailed displacement information allowed us to derive precise physical quantities, notably the strain field energy, from the resolved strain distributions.

The improved imaging enabled us to calculate the normal strain, ɛ_*q*_, at each position along the *q*-direction of the particle, defined as
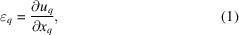
where *u*_*q*_ is the displacement along the *q*-direction and *x*_*q*_ is the position coordinate in that direction. By integrating the strain over the entire volume *V* of the nanoparticle and incorporating the bulk modulus *K*, we calculated the strain field energy, *E*_s_ (Kittel & McEuen, 2018 ▸),
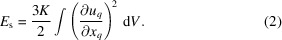
To relate the strain field energy to the voxel size ratio *r* (the ratio of the voxel size to the lattice constant *a*), we expressed the total strain field energy from the imaging results in *Vx* as a function of *r*,
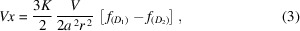
where *V* is the volume of the nanoparticle, *f*_(*x*)_ is a Gaussian function, and *D*_1_ and *D*_2_ represent the average distances between different lattice points within the same voxel and second-neighbor voxels, respectively (details provided in Section S1 of the supporting information).

**Figure 5:**
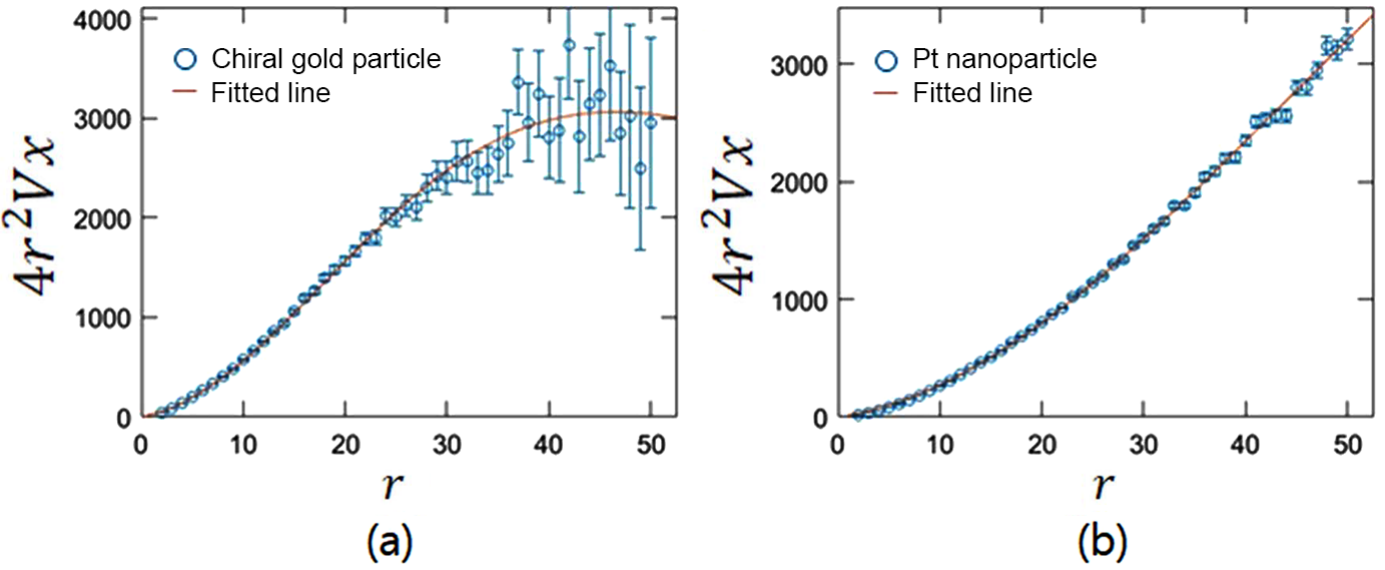
The *Vx* values and regression curves for (*a*) the chiral gold particle and (*b*) the Pt nanoparticle. The blue circles represent the plots of 4*r*^2^*Vx* in terms of *r*, and the red lines show the fitting result using 4*r*^2^*Vx* = 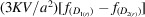.

By plotting 4*r*^2^*Vx* against *r* (as shown in Fig. 5 ▸), we fitted the data to determine the strain field energies for both samples. The calculated strain field energy was 15 fJ for the chiral gold nanoparticle and 10 fJ for the Pt nanoparticle.

### 3.5. Strain correlation length

In determining the actual strain field energy, we derived a function that describes the strain correlation within the nanoparticles. This function is given by
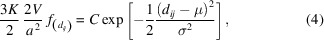
where *d*_*ij*_ is the distance between lattice points *i* and *j*, and 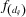 is a Gaussian function representing the correlation of strain between these points. This equation can be expressed in terms of the constants *C*, μ and σ.

For the chiral gold nanoparticle, we obtained results *C* = 4904, μ = −6.4 and σ = 47.8. The strain correlation length is defined as the distance *d*_*ij*_ at which 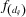 reduces to half of its maximum value *f*_(0)_. Since *f*_(50)_ = 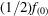, we found that the strain correlation length halves at 50 lattice units for the chiral gold nanoparticle. Similarly, for the Pt nanoparticle, we obtained results *C* = 9518, μ = −12.7 and σ = 113.3. By *f*_(121)_ = 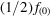, we determined that the strain correlation length halves at 121 lattice units for the Pt nanoparticle.

These results indicate that the strain correlations extend over different length scales in the two nanoparticles, reflecting variations in their internal strain distributions. The shorter strain correlation length in the chiral gold nanoparticle suggests more localized strain variations, possibly due to its complex geometry and the presence of chiral shapes. In contrast, the longer correlation length in the Pt nanoparticle implies a more uniform strain distribution across the particle, consistent with its more straightforward, well defined faceted structure.

By applying ExImRes to both the chiral gold and Pt nanoparticles, we demonstrated that this method can successfully reconstruct nanostructures with varying complexities. The enhanced images allowed for more precise calculations of strain field energy and correlation lengths of atomic deformations, surpassing the limitations of traditional BCDI methods. This validation confirms that ExImRes is a powerful tool for high-resolution analysis in material science applications.

## 4. Conclusion and outlooks

By applying the ExImRes method to the images of BCDI data, we successfully obtained improved resolution images of both the chiral gold nanoparticle and the platinum nanoparticle, achieving nearly a twofold improvement in resolution. This significant enhancement allowed us to extract detailed lattice-scale information, leading to precise calculations of physical quantities such as strain field energy and strain correlation lengths. ExImRes represents an innovative shift in overcoming the spatial resolution limitations of traditional BCDI methods, achieving remarkable results without the need for additional data or changes in the experimental setup. Its ability to provide high-resolution images makes it a valuable tool with widespread potential applications in imaging techniques that involve reciprocal to real space transformations. Looking forward, the implementation of ExImRes could revolutionize nanoscale imaging techniques, enabling more detailed analysis of material properties and facilitating advances in areas like defect analysis, phase transitions and catalysis research.

## Supplementary Material

Supporting information, including Figs. S1 to S4 and Tabes S1 to S3. DOI: 10.1107/S1600577525002942/mo5299sup1.pdf
