# Supplementary material for: Enhancing resolution with the extended image restoration method: strain field energy and correlation length analysis in Bragg coherent X-ray diffraction imaging
Source: J Synchrotron Radiat. 2025 Apr 25;32(Pt 3):743–9. doi: 10.1107/S1600577525002942 (PMC12067331; doi:10.1107/S1600577525002942)
Supplement: Supplementary file 1 [file s-32-00743-sup1.pdf]

JOURNAL OF SYNCHROTRON RADIATION

Volume 32 (2025)

Supporting information for article:

Enhancing resolution with the extended image restoration method: strain field energy and correlation length analysis in Bragg coherent X-ray diffraction imaging

Kyuseok Yun, Sungwook Choi and Hyunjung Kim

S1. Strain field energy of the particle from imaging results

The strain field energy, E_s, is given by the following equation,

$$E_s = \frac{3K}{2} \int \left(\frac{\partial u_q}{\partial x_q} \right)^2 dV$$

where K is the bulk modulus and u_q is the displacement. At the atomic scale, since u_q is not continuous but discrete, the inner part of the integral can be expressed by

$$\left(\frac{\partial u_q}{\partial x_q} \right)^2 = \left(\frac{u_i - u_{i-1}}{a} \right)^2 = \frac{u_i^2 + u_{i-1}^2 - 2u_i u_{i-1}}{a^2}$$

where a is lattice constant and u_i is the displacement of the ith lattice point. Consequently, the integral for the strain field energy, which consists of lattice points, is calculated by

$$\int \left(\frac{\partial u_q}{\partial x_q} \right)^2 dV = a^3 \left(\frac{\sum u_i^2 + \sum u_{i-1}^2 - \sum 2u_i u_{i-1}}{a^2} \right).$$

When dealing with image data composed of voxels, each voxel represents the average information contained within the lattices it encompasses. Denoting the ratio of voxel size to lattice spacing as r, the displacement of the i th voxel (U_i) is given by

$$U_i = \frac{\sum_{k=1}^{r^3} u_k}{r^3}.$$

The strain at each voxel is calculated via the gradient function.

$$\varepsilon_i = \frac{\partial U_i}{\partial x} = \mathrm{grad}(U_i) = \frac{U_{i+1} - U_{i-1}}{2ar}$$

where ε_i is the strain of i th voxel. The integral part computed using this strain expression is

$$\int \left(\frac{\partial U_i}{\partial x} \right)^2 dV = \sum (ar)^3 \left(\frac{U_{i+1} - U_{i-1}}{2ar} \right)^2 = \sum a^3 r^3 \frac{\left(\sum_1^{r^3} u_+ - \sum_1^{r^3} u_- \right)^2}{4a^2 r^8},$$

where u_+ and u_- represent the lattices contained within the voxel U_{i+1} and U_{i-1}, respectively. We needed to introduce a new function. $f_{(d_{ij})}$ to concatenate the results represented by the summation, as the total sum of products between lattices decreases with increasing distance due to decreasing correlation. We defined this function as a decaying Gaussian.

$$\frac{\sum u_i u_j}{n} = f_{(d_{ij})} = Ae^{-\frac{(d_{ij}+c)^2}{2\sigma^2}}$$

where n is the number of terms for Σ, d_{ij} is the distance between lattices expressed by the displacement u_i and u_j, with A, c, and σ as constants. The average distances between lattices within a voxel (d_{homo}) and second-neighbor voxels (d_{hetero}) can be easily computed the RMS values

$$\mathrm{rms}(d_{\mathrm{homo}}) = \sqrt{\frac{\sum_{x=1}^{r}\sum_{y=1}^{r}\sum_{z=1}^{r}\sum_{h=1}^{r}\sum_{k=1}^{r}\sum_{l=1}^{r}(x-h)^2+(y-k)^2+(z-l)^2}{r^6}} = \sqrt{\frac{1}{2}r^2-\frac{1}{2}}$$

$$\equiv D_1 \text{ [lattice]}$$

$$\mathrm{rms}(d_{\mathrm{hetero}}) = \sqrt{\frac{\sum_{x=1}^{r}\sum_{y=1}^{r}\sum_{z=1}^{r}\sum_{h=1}^{r}\sum_{k=1}^{r}\sum_{l=2r+1}^{3r}(x-h)^2+(y-k)^2+(z-l)^2}{r^6}} = \sqrt{\frac{9}{2}r^2-\frac{1}{2}}$$

$$\equiv D_2 \text{ [lattice].}$$

We defined the actual strain field energy possessed by the nanocrystal and the strain field energy calculated from imaging results as Lt and Vx, respectively, to distinguish between them. Incorporating this function, we could simplify Vx as

$$\frac{3K}{2}\int\left(\frac{\partial U_i}{\partial x}\right)^2 dV = \frac{3K}{2}\frac{V}{2a^2r^2}\left(f_{(D_1)} - f_{(D_2)}\right) \equiv Vx$$

where V is the particle volume. Additionally, Lt takes the form.

$$\frac{3K}{2}\int\left(\frac{\partial u_q}{\partial x_q}\right)^2 dV = \frac{3K}{2}\frac{2V}{a^2}\left(f_{(0)} - f_{(1)}\right) \equiv Lt.$$

To find Lt, we could fit $4r^2Vx$, since multiplying Vx by $4r^2$ gave us the same constant term, allowing us to determine the value of Lt.

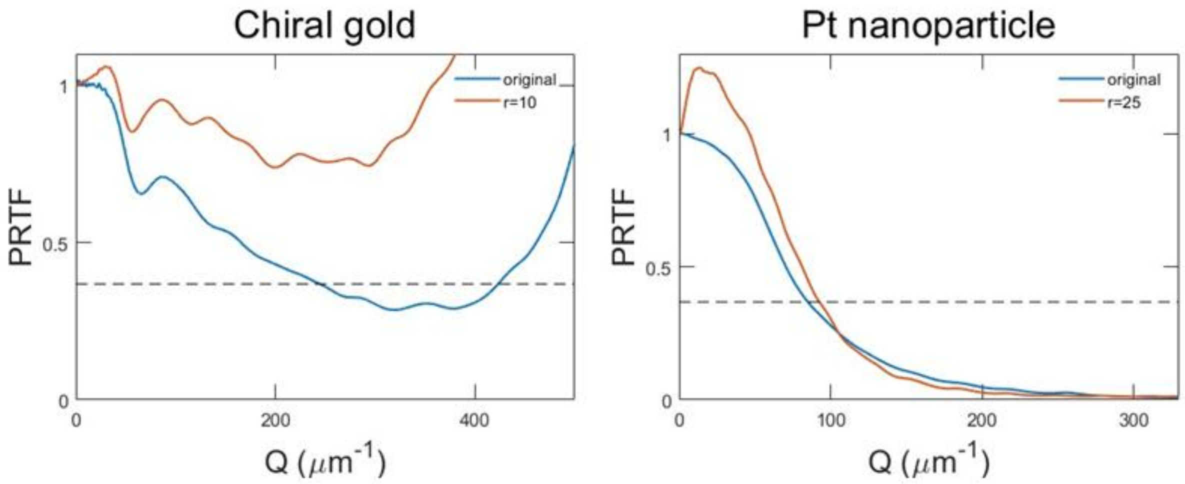

Figure S1 PRTF results for chiral gold and Pt nanoparticles. Using the PRTF, the resolution corresponds to the 1/e value. In the case of chiral gold, the resolution under the original experimental condition is 248 μm^{-1} (~25 nm). However, at r = 10, the PRTF does not reach the 1/e threshold, so the resolution could not be determined. It means that the ExImRes method retrieves the information at large Q in the near-edge regions of the detector, where the information is nearly absent. In the case of Pt, the resolution under the original experimental condition was 85 μm^{-1} (~74 nm), and for r = 25, a resolution of 93 μm^{-1} (~67 nm) was obtained.

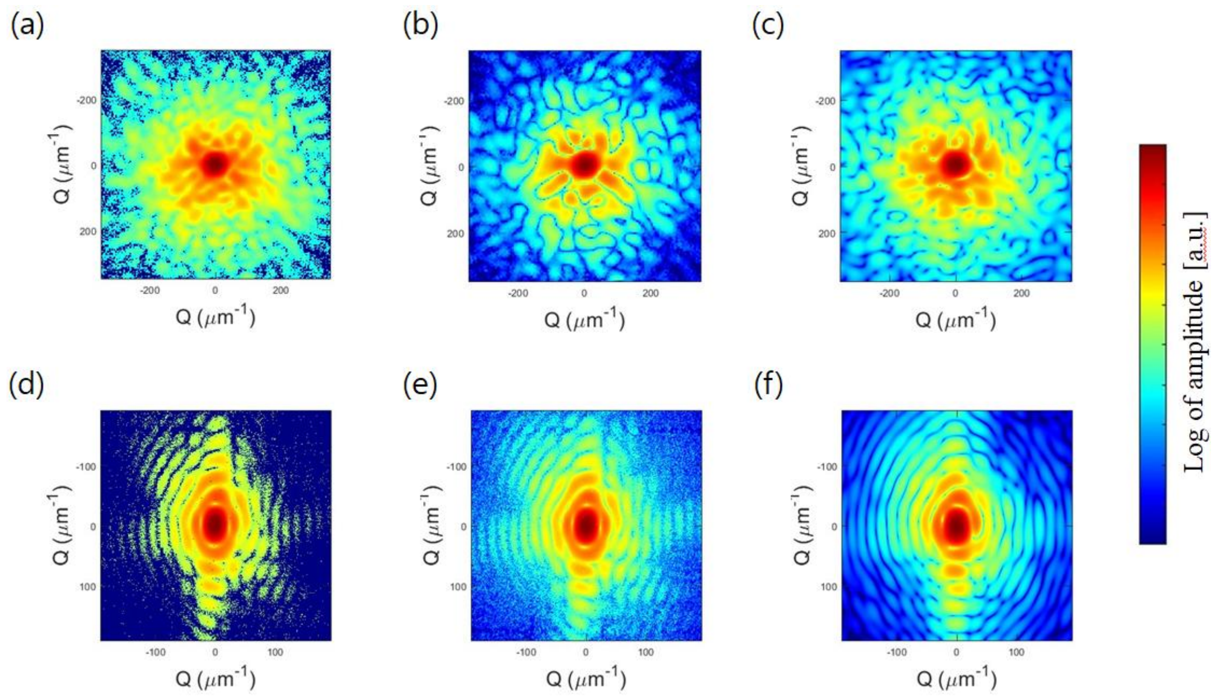

Figure S2 Comparison of Fourier transformed patterns. In the case of chiral gold, we present the center frame of (a) the measured pattern, (b) the averaged diffraction patterns of 10 by Fourier transformation from the images under the original condition, and (c) the average of Fourier-transformed images before merging in the r = 10 case. For the Pt nanoparticle, we show the center frame of (d) the measured data, (e) the averaged diffraction patterns of 10 by Fourier transformation from the images under the original condition, and (f) the average of Fourier-transformed images before merging in the r = 25 case.

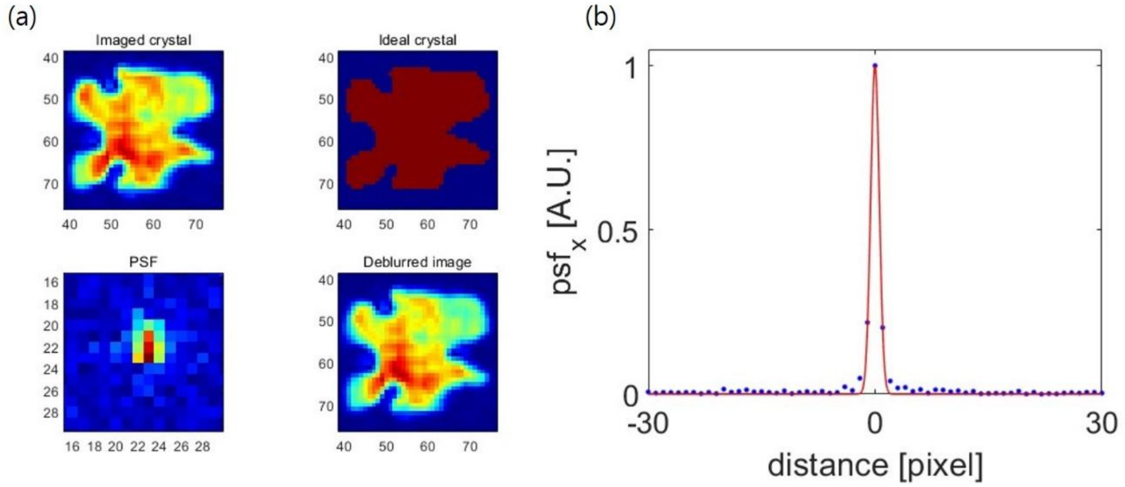

Figure S3 The spatial resolution determined using the point spread function. **(a)** Calculation result of the 3D point spread function by blind deconvolution method. YZ-center slices are shown. **(b)** Typical section of the PSF function and parameter fitting. The full-width-half-maximum of the Gaussian represents the spatial resolution for the corresponding direction.

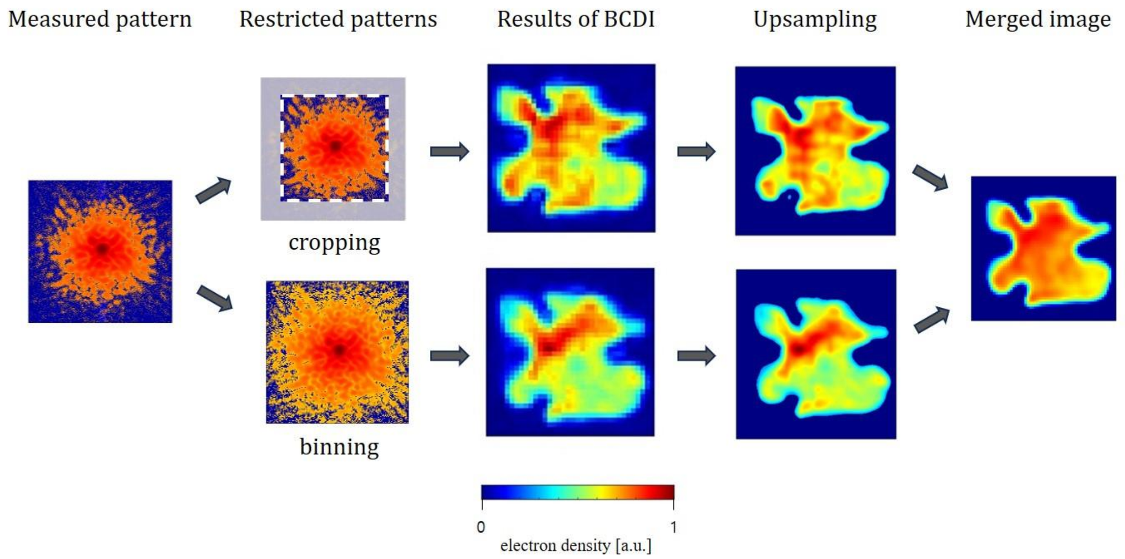

Figure S4 The processing steps for merging individual images are as follows: multiple restricted patterns are generated from the measured pattern using cropping or binning. Then, each pattern is independently imaged using a phase reconstruction algorithm. The next step is that voxel sizes are standardized through upsampling to form a common grid. Finally, the images are aligned by centering at their center of masses, and then they are merged at their proper positions.

Table S1 Resolution parameters for chiral gold particle under the original experimental condition, $r = 2$, and $r = 10$.

**Parameters**	**Original experimental condition**	$\boldsymbol{r = 2}$	$\boldsymbol{r = 10}$
$\boldsymbol{\sigma_x}$	0.5679 pixel	2.2705 pixel	0.5898 pixel
$\boldsymbol{\sigma_y}$	0.5322 pixel	2.8899 pixel	0.5279 pixel
$\boldsymbol{\sigma_z}$	0.4914 pixel	1.4036 pixel	0.5350 pixel
**Spatial resolution**	7.8452 nm	4.2023 nm	5.29037.8452 nm
**Pixel resolution**	6.28 nm/pixel	0.8156 nm/pixel	4.078 nm/pixel

Table S2 This table shows the data obtained based on the constrained manner from the original pattern of the chiral gold particle. ‘Binning’ refers to the number of bins of data, and ‘Size of data’ refers to the sizes of the data in the order of x, y, z dimensions.

Data	Binning	Size of data			r
chiral_001	1	242	456	119	30.80
chiral_002	1	242	456	119	30.80
chiral_003	2	122	229	119	15.40
chiral_004	2	122	229	119	15.40
chiral_005	2	122	230	119	15.40
chiral_006	2	122	230	119	15.40
chiral_007	2	123	229	119	15.40
chiral_008	3	82	153	119	15.40
chiral_009	3	82	153	119	15.40
chiral_010	3	82	153	119	15.40
chiral_011	3	82	153	119	15.40
chiral_012	3	82	153	119	15.40
chiral_013	3	82	153	119	15.40
chiral_014	3	82	153	119	15.40
chiral_015	3	82	153	119	15.40
chiral_016	3	82	153	119	15.40
chiral_017	3	83	153	119	15.40
chiral_018	4	61	115	119	15.40
chiral_019	4	61	115	119	15.40
chiral_020	4	61	115	119	15.40
chiral_021	4	61	116	119	15.40
chiral_022	4	61	115	120	15.40
chiral_023	4	61	115	119	15.40
chiral_024	4	62	115	119	15.40
chiral_025	4	61	116	120	15.40
chiral_026	4	62	115	119	15.40
chiral_027	4	62	115	119	15.40

chiral_028	4	62	115	119	15.40
chiral_029	4	62	116	119	15.40
chiral_030	4	62	115	119	15.40
chiral_031	4	62	115	119	15.40
chiral_032	4	62	115	119	15.40
chiral_033	4	62	116	119	15.40
chiral_034	4	62	115	119	15.40
chiral_035	1	256	256	72	27.44
chiral_036	1	256	256	80	24.70
chiral_037	1	256	256	96	23.10
chiral_038	1	256	256	128	23.10
chiral_039	1	256	256	144	23.10
chiral_040	1	256	256	160	23.10
chiral_041	1	256	256	192	23.10
chiral_042	1	288	288	72	27.44
chiral_043	1	288	288	80	24.70
chiral_044	1	288	288	96	20.58
chiral_045	1	288	288	128	20.53
chiral_046	1	288	288	144	20.53
chiral_047	1	288	288	160	20.53
chiral_048	1	288	288	192	20.53
chiral_049	1	320	320	72	26.40
chiral_050	1	320	320	80	24.70
chiral_051	1	320	320	96	20.58
chiral_052	1	320	320	128	18.48
chiral_053	1	320	320	144	18.48
chiral_054	1	320	320	160	18.48
chiral_055	1	320	320	192	18.48
chiral_056	1	384	384	72	22.00
chiral_057	1	384	384	80	22.00
chiral_058	1	384	384	96	20.58

chiral_059	1	384	384	128	15.44
chiral_060	1	384	384	144	15.44
chiral_061	4	96	96	72	22.00
chiral_062	4	96	96	80	20.58
chiral_063	4	96	96	96	15.44
chiral_064	4	96	96	128	15.40
chiral_065	4	96	96	144	15.40
chiral_066	4	96	96	160	11.55
chiral_067	4	128	128	192	19.21
chiral_068	4	128	128	72	17.29
chiral_069	4	128	128	80	16.50
chiral_070	4	128	128	96	15.44
chiral_071	4	128	128	128	13.72
chiral_072	4	128	128	160	19.21
chiral_073	3	160	160	72	17.60
chiral_074	3	160	160	80	17.60
chiral_075	3	160	160	96	15.44
chiral_076	3	160	160	128	13.72
chiral_077	3	160	160	144	12.35
chiral_078	3	160	160	160	12.32
chiral_079	3	160	160	192	19.55
chiral_080	3	144	144	72	19.55
chiral_081	3	144	144	80	19.55
chiral_082	3	144	144	96	15.44
chiral_083	3	144	144	128	13.72
chiral_084	3	144	144	144	13.69
chiral_085	3	144	144	160	13.69
chiral_086	3	144	144	192	22.00
chiral_087	3	128	128	72	22.00
chiral_088	3	128	128	80	20.58
chiral_089	3	128	128	96	15.44

chiral_090	3	128	128	128	15.40
chiral_091	3	128	128	144	15.40
chiral_092	3	128	128	160	15.40
chiral_093	3	128	128	192	19.21
chiral_094	5	96	96	72	17.60
chiral_095	5	96	96	80	17.60
chiral_096	5	96	96	96	15.44
chiral_097	5	96	96	128	13.72
chiral_098	5	96	96	144	12.35
chiral_099	5	96	96	160	12.32
chiral_100	5	96	96	192	21.12
chiral_101	5	80	80	72	21.12
chiral_102	5	80	80	80	20.58
chiral_103	5	80	80	96	15.44
chiral_104	5	80	80	128	14.78
chiral_105	5	80	80	144	14.78
chiral_106	5	80	80	160	14.78
chiral_107	5	80	80	192	23.46
chiral_108	5	72	72	72	23.46
chiral_109	5	72	72	80	20.58
chiral_110	5	72	72	96	16.42
chiral_111	5	72	72	128	16.42
chiral_112	5	72	72	144	16.42
chiral_113	5	72	72	160	16.42
chiral_114	5	72	72	192	16.42

Table S3 This table shows the data obtained based on the constrained manner from the original pattern of the Pt nanoparticle.

Data	Binning	Size of data			r
Pt_001	1	384	384	128	32.63
Pt_002	1	320	320	128	33.71
Pt_003	1	288	288	128	33.71
Pt_004	1	256	256	128	34.26
Pt_005	1	192	192	128	45.68
Pt_006	1	160	160	128	54.82
Pt_007	1	384	384	96	32.63
Pt_008	1	320	320	96	39.15
Pt_009	1	288	288	96	43.50
Pt_010	1	256	256	96	44.95
Pt_011	1	192	192	96	45.68
Pt_012	1	160	160	96	54.82
Pt_013	2	192	192	128	32.63
Pt_014	2	160	160	128	33.71
Pt_015	2	144	144	128	33.71
Pt_016	2	128	128	128	34.26
Pt_017	2	96	96	128	45.68
Pt_018	2	192	192	96	32.63
Pt_019	2	160	160	96	39.15
Pt_020	2	144	144	96	43.50
Pt_021	2	128	128	96	44.95
Pt_022	2	96	96	96	45.68
Pt_023	1	384	384	80	37.76
Pt_024	1	320	320	80	39.15
Pt_025	1	288	288	80	43.50
Pt_026	1	256	256	80	48.94
Pt_027	1	192	192	80	53.94
Pt_028	1	160	160	80	54.82

Pt_029	3	128	128	128	32.63
Pt_030	3	96	96	128	33.71
Pt_031	3	80	80	128	36.54
Pt_032	3	72	72	128	40.60
Pt_033	3	64	64	128	45.68
Pt_034	3	128	128	96	32.63
Pt_035	3	96	96	96	43.50
Pt_036	3	80	80	96	44.95
Pt_037	3	72	72	96	44.95
Pt_038	3	64	64	96	45.68
